# Axillary Artery Thrombosis in a Neonate *In Utero*: A Case Report

**DOI:** 10.1155/2014/417147

**Published:** 2014-01-28

**Authors:** A. Szvetko, E. Hurrion, A. Dunn, S. Fasihullah, S. Withers

**Affiliations:** ^1^Genesis Clinical Genetics, Suite 5.06 Pindara Specialist Suites, 29 Carrara Street, Benowa, QLD 4217, Australia; ^2^School of Medicine, Faculty of Health Sciences, The University of Queensland, Herston Rd, Brisbane, QLD 4006, Australia; ^3^Dunn Obgyn, Suite 4.08 Pindara Specialist Suites, 29 Carrara Street, Benowa, QLD 4217, Australia; ^4^Leading Steps Paediatric Clinic, Suite 4.05 Pindara Specialist Suites, 29 Carrara Street, Benowa, Qld 4217, Australia

## Abstract

We describe a neonate of 38-week and 6-day gestation born by lower uterine cesarean section for breech presentation, where it was evident on delivery that there was significant edema of the right arm from the deltoid to the distal tips of the fingers. Doppler flow ultrasound revealed extensive arterial thromboembolus. Intravenous heparin was prescribed for three days at a dose of 27.5 U/kg/h, targeting an activated partial thromboplastin time (APTT) of 60–75 seconds, followed by a course of subcutaneous enoxaparin at a dose of 1.8 mg/kg and then 2 mg/kg twice daily, titrated to a factor Xa level of 0.5–1.0 U/mL for another three days. Significant clinical improvement occurred and the child was eventually, discharged on subcutaneous enoxaparin. Magnetic resonance imaging showed multiple intracranial abnormalities. At five months increased upper limb tone, brisk reflexes, and small head circumference were noted. At one year, increased tone and increased paucity of movement on the right side persisted, and some speech delay and visual inattention were noted. Recent follow-up at 16.5 months of age demonstrated a right sided hemiplegia with increased tone and brisk reflexes. We describe the case in detail and review current knowledge regarding the management of arterial thrombosis in the neonate.

## 1. Introduction

Neonatal arterial thrombosis is a serious condition resulting in high rates of morbidity and mortality [1]. The ability to form a blood clot in a controlled manner is essential; however, uncontrolled or excessive clotting may be life-threatening.

The normal formation of a fibrin clot in the body is tightly regulated by anticoagulants. Regularly, local thrombin binds with thrombomodulin located on endothelium activating protein C. Activated protein C along with its cofactor protein S then inhibits factors Va and VIIIa by cleavage, preventing random clot formation. Additionally, antithrombin directly inhibits thrombin and factors IXa, Xa, XIa, and XIIa. The major causes of thrombosis in childhood are introduced venous access devices; the presence of phospholipid antibodies; proteins C and S mutations; factor V (Leiden) mutations; antithrombin deficiency, and less commonly: dysfibrinogenemia; anomalies in the molecular architecture of fibrinogen, and hyperhomocysteinemia, which affects both the vascular wall structure and coagulation constituents [2–4].

Although our understanding of the adult hemostatic system is quite sophisticated, several key differences underlie the structure and function of the neonatal system that make direct therapeutic extrapolation problematic. Differences in synthesis, concentration, and degradation of substituents, as well as regulation of thrombin and plasmin are seen in the neonate [5]. Prematurity is also a known risk factor for developing thrombosis highlighting the difference between the mature and the developing hemostatic systems [6].

Arterial thromboses represent approximately half of all neonatal thromboembolic events [7]. Thromboembolism occurs more commonly in newborns than older children specifically because neonates have decreased concentrations of proteins C and S and antithrombin III [8]. Maternally, factor VIII and von Willebrand factors are known to be elevated during pregnancy preventing hemorrhage and thrombosis, which in turn confer a protective benefit to the fetus, however that protection wanes following birth [9]. These factors combine to make neonatal thrombosis a complex biochemical process.

Failure to identify thromboembolic disease at the time of birth can have severe consequences. Most thrombosis research to date has focussed on venous etiologies rather than arterial [10]. In this report, we describe the presentation and management of a 38-week and 6-day gestation newborn with an arterial thrombosis of uncertain etiology developed *in utero* leading to diffuse neurological injury. We discuss the case and summarize the current state of knowledge regarding management of neonatal arterial thrombosis.

## 2. Case Report

**Figure 1:**
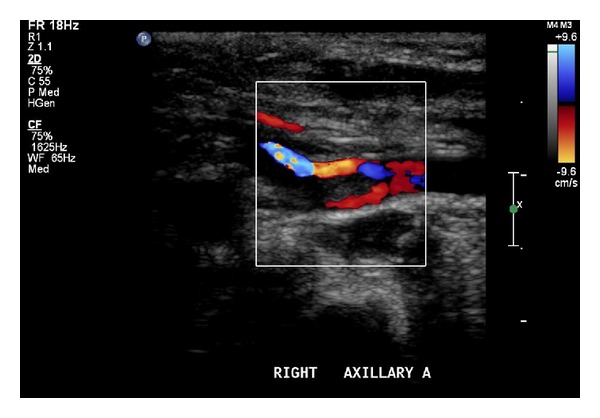
Ultrasound scan of the right axillary artery demonstrating a substantial non-occlusive hypo-echoic thrombus.

**Figure 2:**
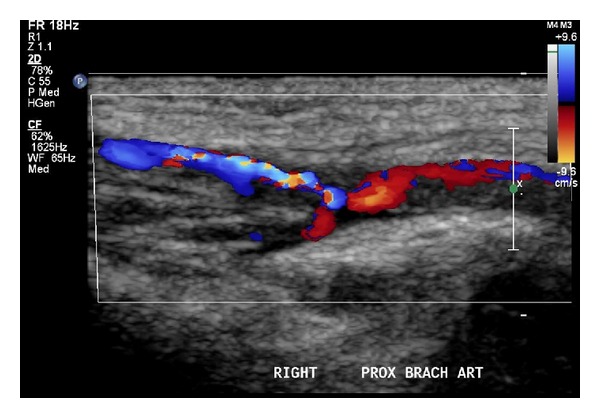
Ultrasound scan of the right proximal brachial artery demonstrating a substantial non-occlusive hypo-echoic thrombus.

We attended a multiparous woman in spontaneous labour at 38 weeks and 6 days gestation who underwent elective lower uterine segment cesarean section for a breech presentation. A baby girl was delivered with a birth weight of 2690 g (10th–25th percentile), length of 46 cm (5–10th percentile), and head circumference of 34 cm (50–75th percentile). Apgar scores were 8 at 1 minute and 9 at 5 minutes. Immediately at delivery, it was evident that there was a significant pitting edema of the right arm, which extended from the deltoid region to the distal tips of the fingers. The right hand was in a flexed position with the hand being positioned under the right mandible. Capillary refill was approximately 4-5 seconds. Brachial and radial pulses were palpable but reduced, and a provisional diagnosis of venous thrombosis was made. The child was subsequently transferred to a special care nursery for further management. Blood tests and coagulation studies were ordered including full blood count, urea and electrolytes, liver function tests, coagulation profile, and D-dimer. Blood test results showed a low platelet count, 66 × 10^9^/L (150–600 × 10^9^/L) with a significantly elevated D-dimer 35.6 mg/L (<0.5 mg/L). Ultrasound scans of the right arm and axilla were performed, demonstrating a non-occlusive hypoechoic thrombus in the distal right and proximal brachial arteries extending for approximately 12 mm (Figures 1 and 2). It was now clear that an arterial thrombosis had occurred. The clot had led to an increased systolic velocity at the level of the thrombus with a flow ratio of 2.9, representing 50% stenosis. Below the thrombus, monophasic flow was demonstrated through the brachial, radial, and ulnar arteries. The decision was made to transfer the child to a tertiary neonatal centre for further management.

Heparin sodium infusion was administered intravenously for three days at a dose of 27.5 U/kg/h, which brought the APTT to within the therapeutic range 60–75 seconds within one day, and some clinical improvement was seen with reduction in swelling improved capillary refill of 3-4 seconds, and stronger peripheral pulses. Days two and three showed further clinical improvement with further reduction in swelling, and yet again stronger pulses. Heparin infusion was ceased on day three in favour of subcutaneous enoxaparin 5 mg (1.8 mg/kg) 12 hourly until day six, at which time the dose was adjusted to 5.5 mg (2 mg/kg) 12 hourly in light of factor Xa levels. Therapy was monitored with regular measurement of anti-Xa levels taken by venous sampling 4 hours after dosing. The therapeutic range of 0.5–1.0 U/mL was targeted and maintained. Discharge planning included the parents administering 14 days of subcutaneous enoxaparin 1.5 mg/kg twice daily. In summary, clinical improvement on heparin sodium infusion was seen and continued from day one to day three, further significant improvements were seen on subcutaneously administered enoxaparin from day three to day six, and adjustments were made for ongoing treatment with subcutaneous enoxaparin for another 14 days after-discharge from the tertiary neonatal centre.

**Figure 3:**
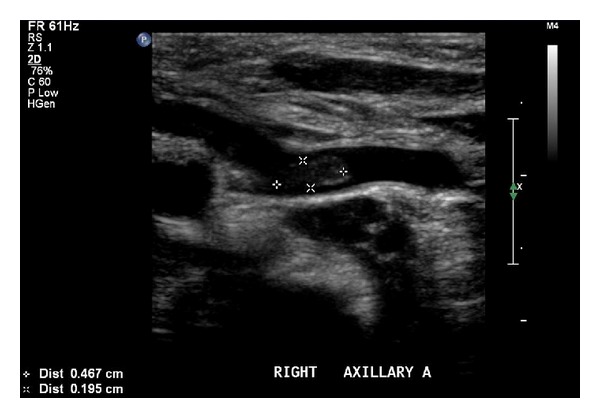
Repeated ultrasound scan showing almost complete resolution of the distal axillary artery thrombus.

**Figure 4:**
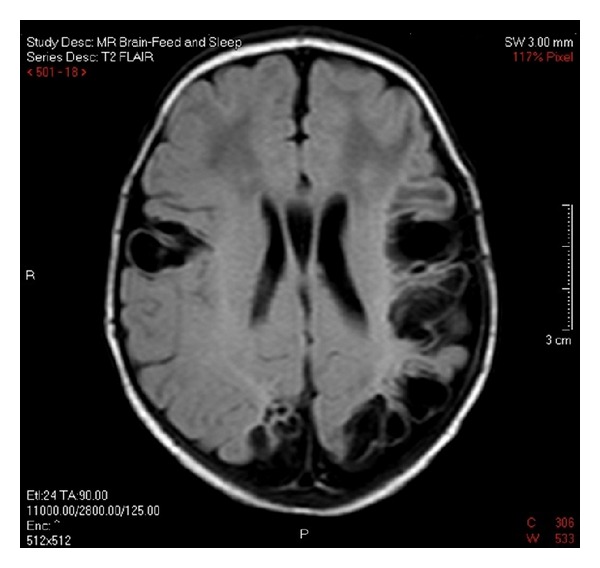
T2-FLAIR imaging four weeks postpartum showing significant lesions at the left parieto-occipital area and to a lesser extent on the right.

**Figure 5:**
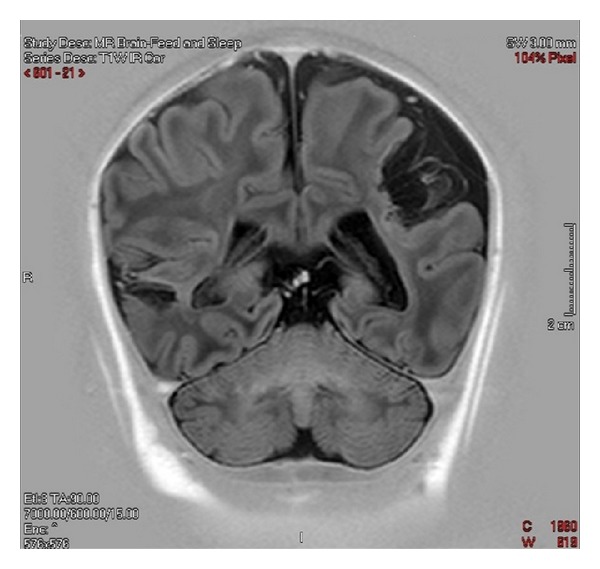
T1-weighted imaging four weeks postpartum showing significant lesions at the left parieto-occipital area.

Further testing was carried out in an attempt to determine a possible cause for the thromboses. Tests included studies for factor V Leiden mutation, lupus antibody, and protein C, protein S, and antithrombin III deficiencies. Peripheral blood sample was sent for thrombophilia mutation screening, and the patient was determined to be normal for the two most common inherited causes of thrombophilia, factor V G1691A mutation, and the prothrombin G20210A mutation. There was no evidence of lupus anticoagulant, and a basic thrombophilia screen was within normal limits: protein C, 48% (17–53%); free protein S 42% (12–60%); and arterial to venous platelet contractile force ratio, 5.8 (>2.8). A repeated ultrasound scan five days after discharge showed almost complete resolution of the distal right axillary artery thrombus, measuring 6 mm in length and 1 mm in depth, without any new thrombus formation (Figure 3). Magnetic resonance imaging (MRI) scans were ordered due to the risk of cerebral embolization. A feed and sleep study was performed imaging axial T1 and T2-weighted, FLAIR and diffusion, sagittal T1 3D gradient echo, and coronal T1 sections. The MRI scan was undertaken four weeks postpartum and showed multiple intracranial abnormalities including gliosis and loss of both white and grey matter in and around the middle and posterior cerebral arteries bilaterally, with particularly significant involvement at the left parieto-occipital area and lesser involvement on the right (Figures 4 and 5). The parenchyma supplied by the anterior cerebral artery appeared normal. The basal ganglia appeared unaffected; however, a negative mass effect was noted at the trigone of the left lateral ventricle due to presumed loss of the adjacent cerebral tissue. Thinning of the *corpus callosum* was evident due to apparent loss of white matter. The midline structures appeared normal otherwise and within normal limits with preservation of the posterior pituitary bright spot with no evidence of Chiari malformation at the cerebellum. The overall impression was that widespread cerebral gliosis primarily involving the parenchyma supplied by the middle cerebral arteries and posterior cerebral arteries bilaterally had occurred.

At 2 months the child was examined; weight was 4.17 kg (3rd–15th percentile), head circumference was 35.4 cm (<5th percentile), and all limbs were being moved independently with no evidence of increased tone. Power, tone, and sensation all appeared within normal limits. A review was also performed at 3 months, where she appeared to be well and thriving; had been feeding 3-hourly, sleeping well, toileting regularly; and was recorded to be 4.82 kg (3rd–15th percentile) with head circumference of 36.1 cm (<5th percentile). Some decreased movement on the right was noted at that time. She was again recently assessed at 5 months of age with no fresh concerns and was noted to be 6.4 kg (15th–50th percentile), head circumference have 38.1 cm (<5th percentile); and be bright, alert, and interactive. There appeared to be no visual deficits although significant occipital tissue loss had been previously demonstrated on MRI scan. Marginally increased upper limb tone was noted on that occasion with brisk reflexes and down-going plantar reflexes bilaterally. Neurological examination was otherwise unremarkable and the child examined normally except for head circumference (<5th percentile). Most recent assessment was at 16.5 months of age at our Growth and Development Clinic. Weight was 9.61 kg (>10th centile), length was 80 cm (<75th centile), and head circumference was 42.5 cm (<3rd centile). The review demonstrated some difficulties with gross and fine motor skills, ongoing right-sided hemiplegia, and persistent increased tone and reflexes but otherwise normal systemic examination. Cognitive development, assessed on the Bayley III Scales of Infant and Toddler Development, was normal (DQ 95). Further physiotherapy, occupational therapy, and pediatric neurology follow-up have been recommended. We plan to review the child's progress again at the two-year mark not withstanding any interim changes or concerns.

## 3. Discussion

Neonatal thromboembolism carries a high risk of both mortality and serious sequelae [11]. Thromboses can impair circulation and may commonly result in damage to the arms, legs, lungs, kidneys, heart, brain, or intestines. The clinical presentation depends on the timing and location of the initial lesion and may be overlooked unless there is a heightened index of suspicion. Thrombus formation in the neonate often occurs adjunct to the normal process of coagulation. Activation of the extrinsic (tissue factor) or intrinsic (intravascular) pathway results in precipitation of fibrin at the site of the lesion. Thrombocytes adhere to and fibroblasts migrate to the formed clot, and fibrin molecules then interlink. Data regarding the safe and efficacious use of low molecular weight heparin in neonates is scarce [12]. The most common treatments are watchful waiting; heparin; or clot-dissolving drugs such as streptokinase, urokinase, or tissue plasminogen activator. Thrombolytics must be used with caution because they may precipitate severe bleeding. Although there are various therapeutic agents, previous review of the medical literature found that no randomized controlled trials comparing clot-dissolving drugs in neonates exist [13]. Furthermore, long-term follow-up in neonates is also limited.

Thrombophilia has previously been associated with a variety of adverse pregnancy outcomes including recurrent fetal loss, intrauterine growth retardation, *abruptio placentae*, and preterm delivery [14]. The etiology of neonatal thrombosis may be either maternal or fetal. In terms of maternal etiology it is plausible that placental embolic showers may contribute in a significant way to embolic events such as that reported here. Indeed, the physiological changes that occur during pregnancy that lead to a hypercoagulable state are well described [15]. Embolic showers from the placenta may explain brachial plexus and carotid clots such as that seen in this case. Although seldom reported, placental embolic showers may be associated with such events. Although MRI scans as well as tests for hypercoagulability may be negative, they are important prognostically as they demonstrate that the risk of recurrent stroke and inheritance of the pathology is unlikely.

In the current case, it is difficult to postulate the precise origin of the embolus; however, it is most likely to have originated in the placenta, traversed the umbilical vein, and then travelled into the fetal circulation. More specifically, it appears that the clot had travelled up through the liver and inferior *vena cava* into the right ventricle, crossed into the left ventricle, and then had subsequently showered up into the right axillary artery and into the carotids. At the time of the baby's delivery, there was nothing special or specific with respect to the umbilical cord to suggest cord abnormality or malformation. On consideration of the neurological implications, it appears that a clot had travelled via the carotids to the Circle of Willis then to the cerebrum resulting in ischemia affecting the middle cerebral and posterior cerebral artery territories. Indeed, this was well demonstrated on MRI studies four weeks after birth. As a cautionary note, an initial head ultrasound that was undertaken within 24 hours of delivery was reported as showing normal cerebral parenchymal echogenicity. There was thought to be slight asymmetry in the lateral ventricles with the left being a little larger than the right; however, there was no evidence of hydrocephalus or intracranial hemorrhage. The posterior fossa structures and extra axial cerebrospinal spaces were reported as normal, and the superior sagittal sinus was patent. It is of interest to note that the initial head ultrasound done at around 24 hours of age was reported as essentially normal. This may prove to be an important consideration in similar cases. Initial methods for diagnosis include a thorough but timely physical examination and Doppler ultrasound scan with angiography, which is considered the gold standard diagnostic test; however, this method may in itself increase the risk of thrombosis, so it must be employed judiciously [16]. The interpretation of D-dimers, although routinely ordered, should be interpreted cautiously as they vary greatly between even normal infants [17].

Antithrombotic therapy in neonates is challenging owing to altered metabolism of anticoagulants. Furthermore, only limited guidelines exist, and those have been extrapolated from adult observations [18]. Given the differences in bleed risk in neonates and the lack of long-term follow-up data, the therapeutic decision can seem rather challenging. The therapeutic options commonly available include unfractionated heparin, which is cleared faster in neonates due to increased volume of distribution but necessitates venous access and ongoing frequent monitoring [19]; low molecular weight heparin, which is also cleared faster in neonates (the most common of which are enoxaparin and reviparin), which facilitate a more predictable pharmacokinetic profile and require minimal monitoring, have easier subcutaneous administration, and carry lower risk of osteopenia than unfractionated heparin [20]; and oral anticoagulants, warfarin, which is problematic due to the route of administration as well as interactions with fortified formula which makes neonates more resistant to the drug [16]. Other therapeutic options include thrombolytic therapy with streptokinase, urokinase, and tissue plasminogen activator, which have shown variable efficacy with disparate practices in administration [21]. Some studies advocate the use of tissue plasminogen activator rather than streptokinase or urokinase, restricting administration to newborns with life, organ, or limb threatening events only, due to the risk of major bleeding [22]. Recent research has examined the role of prourokinase, recombinant staphylokinase, and alfimmprase as fibrinolytics for peripheral artery occlusion. In those studies it appears that although intra-arterial recombinant tissue plasminogen activator appears more effective than streptokinase and urokinase even more so, the general paucity of data does not yet allow for clear conclusions to be drawn [23]. A recent Cochrane review examined surgery versus thrombolysis for the initial management of acute limb ischemia in order to determine whether physical intervention outperforms thrombolytic therapy. The results of that review indicate that there is no overall difference in limb salvage or death at one year between surgery and thrombolysis, although it is not yet clear whether long-term neurological outcomes are improved. The risks of surgery must be considered on a case-by-case basis, with possible indications being compartment syndrome, which would necessitate an emergent fasciotomy to salvage a limb [24].

Thromboembolic disease in neonates requires prompt diagnosis and treatment to avert potentially serious complications. A high index of suspicion should be adopted in at-risk cases. Clinical examination and Doppler ultrasound with or without angiography remain the choice method for detection.

Current therapies involve the empirical administration of antithrombolytic medications including unfractionated heparin, low molecular weight heparin, enoxaparin and reviparin, and tissue plasminogen activator; however, caution should be exercised in the administration of those drugs as their profiles have been extrapolated from adult observations, and their use carry an increased bleed risk in the newborn. The surgical option should be reserved for severe and emergent cases. The pharmacokinetics of the discussed medications is vastly different in the neonate and there is little objective evidence to date regarding the best long-term outcome. Further studies regarding the safety and efficacy of these and other potential therapies could facilitate the determination of a more favorable approach to deal with thromboembolic events in pediatric cases. In the current report a conservative approach employing the administration of heparin sodium IV for three days (27.5 U/kg/h, target APTT 60–75 seconds), followed by enoxaparin sodium SC for another three (1.8 mg/kg 12-hourly) and then 14 days (2 mg/kg 12-hourly), keeping the target factor Xa level to 0.5–1.0 U/mL, demonstrated good thrombolysis without adverse effects.

The most important lesson from this case is the unexpected cerebral involvement. The Thrombosis and Hemostasis in Newborns (THiN) group does not include neuroimaging in their algorithm of non-CNS thrombosis [1], yet the underlying aetiological theory makes multiple emboli entirely plausible, and cerebral emboli may not be symptomatic. Hemiplegia is more common and more severe among children with MRI evidence of ischaemic stroke who do not present in the neonatal period [25, 26]. Additionally, the reported sensitivity of ultrasonographic imaging to diagnose cerebral ischemia in the first few days is 68% (though it does improve to 87% on days 4–10) [27]. Therefore, in cases of non-CNS perinatal thromboembolism, even in the absence of seizures or other neurological signs, we would recommend neuroimaging, preferably by MRI.
